# Anthelmintic Efficacy of Palmarosa Oil and Curcuma Oil against the Fish Ectoparasite *Gyrodactylus kobayashii* (monogenean)

**DOI:** 10.3390/ani12131685

**Published:** 2022-06-30

**Authors:** Shun Zhou, Qiuhong Yang, Jing Dong, Yongtao Liu, Ning Xu, Yibin Yang, Xiaohui Ai

**Affiliations:** 1 Yangtze River Fisheries Research Institute, Chinese Academy of Fishery Sciences, Wuhan 430223, China; zhoushun@yfi.ac.cn (S.Z.); yangqh@yfi.ac.cn (Q.Y.); dongjing@yfi.ac.cn (J.D.); liuyt@yfi.ac.cn (Y.L.); xuning@yfi.ac.cn (N.X.); yyang@yfi.ac.cn (Y.Y.); 2 Key Laboratory of Control of Quality and Safety for Aquatic Products, Ministry of Agriculture, Beijing 100071, China

**Keywords:** anthelmintic efficacy, palmarosa oil, curcuma oil, *Gyrodactylus kobayashii*, anesthetic effect

## Abstract

**Simple Summary:**

Aquaculture is an important source of animal protein. However, in recent years, with an intensification of farming, the risk of infectious diseases is increasing, especially the diseases caused by monogeneans, resulting in huge economic losses to aquaculture. Currently, the prevention and control of monogenean in fish mainly rely on some chemical drugs, such as formaldehyde, rotenone, and praziquantel. However, prolonged and frequent use of these chemicals leads to the occurrence of drug resistance, some adverse environmental impacts, and even contamination of fish products with drug residues. Therefore, it is urgent to develop efficient and environment-friendly drugs for the control of monogeneans in aquaculture. Natural plant-derived medicines are one of the alternative options. Therefore, in this study, anthelmintic efficacy of various essential oils was evaluated. The results indicated that palmarosa oil and curcuma oil showed potent anthelmintic activity against monogenean, and curcuma oil also had an anesthetic effect on monogenean. Moreover, these two essential oils displayed a high safety for fish. Therefore, palmarosa oil and curcuma oil could be viable alternatives for the prevention and control of monogenean infection in aquaculture.

**Abstract:**

Monogeneans are a serious threat to the development of aquaculture due to the severe economic losses they cause. The prevention and treatment of this disease are increasingly difficult because of the environmental and health concerns caused by the use of chemical anthelmintics and the emergence of drug resistance. It is thus necessary to search for effective alternatives for the treatment of monogenean infections. In the current study, anthelmintic efficacy of 16 selected essential oils (EOs) was investigated using the goldfish (*Carassius auratus*)–*Gyrodactylus kobayashii* model. The screening experiment indicated that palmarosa oil and curcuma oil had satisfactory anthelmintic activity against *G. kobayashii* with EC_100_ values of 10 and 12 mg/L after 24-h exposure, respectively. The in vivo and in vitro assays indicated anthelmintic efficacy of palmarosa oil against *G. kobayashii* was in a time and dose-dependent manner. Interestingly, curcuma oil showed an anesthetic effect on *G. kobayashii*, and its anthelmintic activity was dose-dependent rather than time-dependent in the concentration range tested in this study. Additionally, the 24-h LC_50_ (50% lethal concentration) against goldfish of these two EOs was 8.19-fold and 5.54-fold higher than their corresponding EC_50_ (50% effective concentration) against *G. kobayashii*, respectively. Moreover, exposure to these two EOs at 100% effective concentration against *G. kobayashii* had no serious physiological and histopathological influence on goldfish. These results demonstrated a high safety for goldfish of these two EOs. Overall, palmarosa oil and curcuma oil could be potential candidates for the treatment of *G. kobayashii* infections in aquaculture.

## 1. Introduction

Monogenean is a widespread ectoparasite of fish and is capable of infecting plenty of wild and cultured fish, for instance, *Gyrodactylus salaris* on Atlantic salmon (*Salmo salar*), *Dactylogyrus vastator* on goldfish (*Carassius auratus*), and *Bychowskyella pseudobagri* on yellow catfish (*Pelteobagrus fulvidraco*); these parasite infections pose a great threat to the survival of wild fish and the sustainable development of aquaculture [1,2]. Gyrodactylids are one of the most common monogeneans mainly inhabiting the skin, gills, and fins of fish [3]. These viviparous monogeneans have a direct life cycle and contain one or more developing embryos in utero in a “Russian-doll” manner [4]. The parasites are attached to the host’s epithelium and feed on mucus and epithelial cells, which would cause a range of clinical symptoms including excessive mucus and localized ulcerations. Heavy infections with gyrodactylids also cause severe fin erosion resulting from localized hyperplasia, necrosis, and loss of epithelial cells on the fins, which greatly reduces the ornamental value of some ornamental fish [5]. Additionally, the lesions caused by the parasites facilitate the invasion of secondary infections with bacteria, fungi, and other parasites, which would increase host morbidity and mortality [6,7]. For example, *G. kobayashii* is a common monogenean ectoparasite, and infection with this parasite resulted in high mortality of goldfish [6].

A variety of substances have been evaluated and used to minimize damage from monogenean infections in aquaculture, including mebendazole, formalin, rotenone, and aqueous aluminum [8,9]. However, these substances exhibited unsatisfactory anthelmintic activity and high toxicity to fish. For example, formalin, a commonly used insecticide in aquaculture, showed 100% anthelmintic efficacy against *G. kobayashii* under laboratory conditions [10], but it did not eliminate gyrodactylids completely under large-scale aquaculture conditions [11]. Additionally, formalin has been classified as a “known carcinogen” by the International Agency for Research on Cancer, and its application in food products is banned [12]. Moreover, prolonged and frequent use of these chemicals led to the occurrence of drug resistance, some adverse environmental impacts, and even contamination of fish products with drug residues [8]. It is thus necessary to find better strategies to control monogenean infections in aquaculture.

Natural or plant-derived herbal products and their derivatives have recently gained more attention as an alternative method for monogenean management [13,14]. Essential oils (EOs) are the secondary metabolites of herbal medicines, and some EOs have been reported to be effective in eliminating monogenean infections [15,16,17,18]. For example, the EO of lemongrass (*Cymbopogon citratus*) showed 100% anthelmintic efficacy against monogenean parasites of the tambaqui (*Colossoma macropomum*) in the in vitro assays [19]. Similar results have also been reported for the EO of shell ginger (*Alpinia zerumbet*) [20]. Moreover, the EOs from three plants of the genus *Piper* (Piperaceae) have been found to possess in vitro anthelmintic activity against monogeneans [21]. Therefore, the use of natural EOs or their molecules may provide a promising alternative for controlling monogenean infections in aquaculture.

In the present study, anthelmintic efficacy of 16 EOs against *G. kobayashii* was investigated using the goldfish-*G. kobayashii* model. The in vitro and in vivo anthelmintic activity of palmarosa oil and curcuma oil, which showed promising anthelmintic activity, were also evaluated. Moreover, the physiological and histopathological effects of these two EOs on goldfish after bath treatment were studied.

## 2. Materials and Methods

### 2.1. Chemicals

Eucalyptus oil and dimethyl sulfoxide (DMSO) were obtained from Shanghai Macklin Biochemical Co., Ltd. (Shanghai, China). Fifteen other essential oils (Table 1) were purchased from Shanghai Yuanye Bio-Technology Co., Ltd. (Shanghai, China). All essential oils were dissolved in DMSO with a concentration of 100 mg/mL and then stored at 4 °C until use. Tricaine methanesulfonate (MS222, Sigma-Aldrich, Saint Louis, MO, USA) was obtained from Jiecheng Yuheng Technology Co., Ltd. (Wuhan, China).

### 2.2. Fish and Parasite

A batch of goldfish with a mean bodyweight of 4.82 ± 0.54 g was obtained from a local commercial fish farm in the city of Wuhan, China. The morphology and swimming behavior of goldfish were observed, and only goldfish with normal swimming behavior and no obvious lesions were selected. These fish were transported to the laboratory in Yangtze River Fisheries Research Institute, Chinese Academy of Fishery Sciences (Wuhan, China) and reared in several 500-L polyethylene tanks supplied with constant aeration (water temperature, 21.3 ± 0.8 °C; pH, 6.9–7.3; dissolved oxygen, 6.3 ± 0.7 mg/L). These fish were fed twice daily with pellet feed, and fish feces and food residues were periodically removed. After two weeks of acclimatization, a series of three successive baths with 30 mg/L of formalin solution was administered to goldfish to remove ectoparasites. After a month of recovery, these ectoparasite-free goldfish were cohabited with *G. kobayashii*-infected goldfish conserved in our laboratory to obtain more infected fish for further experiments.

### 2.3. In Vivo Screening of 16 EOs

According to the methods described by Zhou et al. [22], a preliminary screening experiment was conducted using in vivo assays to identify the minimum effective concentration of the EOs against *G. kobayashii*. In short, a moderately infected goldfish (40–200 worms/fish) were randomly selected and placed into a plastic tank with 0.5 L of dechlorinated water. Then different volumes of stock solutions of EOs were added into plastic tanks to achieve the desired concentrations. Two control groups were as follows: control 1 (0.5 L of dechlorinated water without drugs); control 2 (0.5 L of dechlorinated water with 0.1% DMSO). All treatment and control groups were performed in ten replicates at 21.3 ± 0.8 °C under a photoperiod of 12/12 (light/dark). The number of *G. kobayashii* on the caudal fin of experimental goldfish (after anesthetized with 0.02% MS-222) was counted at 0 and 24 h post-treatment under a stereomicroscope and anthelmintic efficacy of each EO was calculated based on the methods described by Zhou et al. [10]. The EOs with anthelmintic potential were then selected for further study based on anthelmintic efficacy, safety, and dosage.

### 2.4. Anthelmintic Efficacy of Selected EOs

#### 2.4.1. In Vitro Assay

The in vitro assays were conducted according to the methods described by Tu et al. [23] with minor modifications. A *G. kobayashii*-infected goldfish with a high parasite load was selected, and the caudal fin was clipped and cut into tiny pieces. The fin pieces with 2–10 worms were selected and transferred to a 24-well plate, and each well contained 400 µL of dechlorinated water. After that, the number and survival of worms in each well were examined and recorded, and the worms that were dead or dying were not included in the final data analysis. Afterward, each well was added with 100 µL of various concentrations of stock solutions to reach the specified concentrations (10, 20, and 30 mg/L of palmarosa oil; 12, 18, and 24 mg/L of curcuma oil). The wells with 0.03% DMSO were set as the control group. Each treatment was tested in 18 parallel wells with a minimum of 80 worms. After the addition of stock solutions, these worms were continuously monitored every one hour for eight hours under a stereomicroscope. The worms were considered as dead if they failed to respond to slight stimulation by an insect pin and the time of death was also recorded.

#### 2.4.2. In Vivo Assay

The in vivo anthelmintic assays of selected EOs were performed according to the method described in Section 2.3. A total of 110 plastic tanks (15 × 13 × 8 cm) were used, each containing a randomly selected infected goldfish and 0.5 L of dechlorinated water (10 replicates per treatment). After that, the stock solutions of palmarosa oil and curcuma oil were added to each tank, and the experimental concentrations of these two EOs were 0 (control, 0.03% DMSO), 4, 6, 8, 9, 10 mg/L, and 4, 6, 8, 10, 12 mg/L, respectively. The environmental conditions and settings were the same as those described in Section 2.3. The parasite load of each goldfish was counted at 0, 2, and 24 h post-exposure, and the anthelmintic efficacy of EOs was calculated after 2 and 24-h bath treatment.

### 2.5. Acute Toxicity Tests

Acute toxicity against goldfish of the selected EOs was evaluated using a 48-h aqueous static bioassay. The goldfish used in the acute toxicity tests have been reared in the laboratory for more than a month, and only these goldfish with normal swimming behavior and no ectoparasites were selected. Ten goldfish were placed into a 10-L tank with 6 L of dechlorinated water and then exposed to different concentrations of EOs. According to the results of the pre-experiment, the final concentrations of palmarosa oil were set to 30, 35, 40, 45, and 50 mg/L; the final concentrations of curcuma oil were set to 20, 25, 30, 35, and 40 mg/L. Dechlorinated water without drugs was used as a negative control and 0.2% DMSO was used as a positive control. Three replicate aquariums were used for all treatment groups, and a total of 36 aquariums were used. The water temperature was maintained at 21.5 ± 0.4 °C under a photoperiod of 12/12 (light/dark). After a 48-h continuous exposure, dead fish were removed and recorded, and the lethal concentration of each EO was analyzed.

### 2.6. The Physiological and Histological Effects of Eos on Goldfish

Forty-five normally swimming and ectoparasite-free goldfish (mean bodyweight of 11.66 ± 1.62 g) were randomly assigned to nine 52 × 41 × 31 cm plastic tanks with gentle aeration, each tank containing five goldfish and 30 L of dechlorinated water. These goldfish were exposed to 10 mg/L of palmarosa oil and 12 mg/L of curcuma oil (24-h EC_100_ against *G. kobayashii*) for 96 h, and 0.2% DMSO was used as a control. Three replicate tanks were used for each group. The identical environmental conditions were used as those described in the acute toxicity test. These goldfish were fed every day, and only food residues and feces were removed and no water in each tank was renewed. After 96-h exposure, five goldfish from each treatment were randomly selected and anesthetized with MS-222, and blood samples (*n* = 15, 5 samples per treatment) were collected from the caudal vein using syringes with EDTA as an anticoagulant. Subsequently, these goldfish were killed and the gills (*n* = 15, 5 samples per treatment) were immediately removed and fixed in Bouin solution for histological analysis.

These collected blood samples were used for routine hematologic evaluation, and the hematological indicators were determined using an automatic blood cell analyzer (Mindray BC-2800Vet, Shenzhen, China), including red blood cells (RBCs), hemoglobin concentration (Hb), hematocrit (HCT), mean corpuscular volume (MCV), mean corpuscular hemoglobin (MCH), mean corpuscular hemoglobin concentration (MCHC) and platelet (PLT). For histological analysis, the fixed gill tissues were processed and stained with hematoxylin and eosin (H&E) according to routine histological procedures. Subsequently, the stained sections were observed and photographed under a Primo Star microscope (Carl Zeiss Microscopy GmbH, Oberkochen, Germany), and then the pathological changes were analyzed.

### 2.7. Statistical Analysis

The survival analysis of *G. kobayashii* was conducted using the Kaplan–Meier method. The EC_50_, EC_90_, and LC_50_ (50% and 90% effective concentration against *G. kobayashii*; 50% lethal concentration against goldfish) with 95% confidence levels were calculated by Probit analysis. The difference between groups was analyzed by one-way analysis variance (ANOVA). All statistical analyses were conducted using SPSS 20.0 software (IBM Corp., Armonk, NY, USA) and a *p*-value of less than 0.05 was considered statistically significant.

## 3. Results

### 3.1. In Vivo Anthelmintic Efficacy of 16 EOs

The in vivo screening experiment indicated that seven EOs, namely palmarosa oil, curcuma oil, cablin patchouli oil, zedoary oil, rue oil, tea tree oil, and neem oil, were capable of removing gyrodactylids infection without causing the death of fish (Table 1). In particular, the performances of palmarosa oil and curcuma oil were the most satisfactory, showing 100% anthelmintic activity at low concentrations; therefore, these two EOs were selected for further experiments. Cassia oil and eucalyptus oil also showed good anthelmintic activity, reaching 94.57% and 90.84% efficacy at concentrations of 14 and 100 mg/L, respectively, although these concentrations caused the death of fish. The remaining seven EOs displayed weak anthelmintic activity (less than 50% efficacy) and high toxicity to the host; for example, origanum oil, anise oil, and lemon oil at the concentration of 10 mg/L resulted in the death of the host.

### 3.2. Anthelmintic Efficacy of Selected EOs

**Figure 1:**
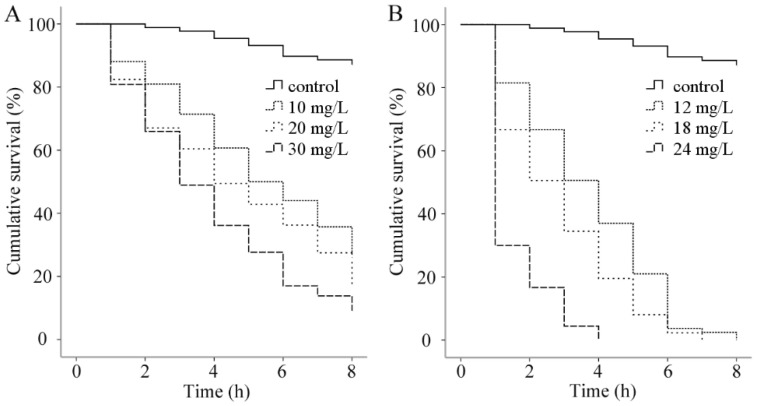
The cumulative survival (%) in vitro of *Gyrodactylus kobayashii* after exposure to different concentrations of palmarosa oil (**A**) and curcuma oil (**B**); 0.03% DMSO (Eucalyptus oil and dimethyl sulfoxide) was used as the control. Parasite death was examined every one hour during 8-h exposure.

The results of in vitro assay of palmarosa oil and curcuma oil against *G. kobayashii* are shown in Figure 1. In the palmarosa oil treatment group, the survival of *G. kobayashii* was negatively correlated with the concentrations of this EO, and there was a significant difference in the survival curves of *G. kobayashii* between palmarosa oil-exposed groups and control group (*p* < 0.01). After 8-h exposure to palmarosa oil in vitro, the mortality of *G. kobayashii* was 73.81, 83.52, and 91.49% at concentrations of 10, 20, and 30 mg/L, respectively. Similar results were observed for the survival of *G. kobayashii* in vitro after treatment with curcuma oil; this EO appeared to be able to kill *G. kobayashii* in a shorter time, resulting in 100% mortality of the worms at 12 mg/L for 8-h exposure, 18 mg/L for 7-h exposure, and 24 mg/L for 4-h exposure, respectively. The cumulative survival of *G. kobayashii* in the control group (0.03% DMSO) reached 95.45 and 90.48% after 4 and 8-h exposure, respectively.

**Figure 2:**
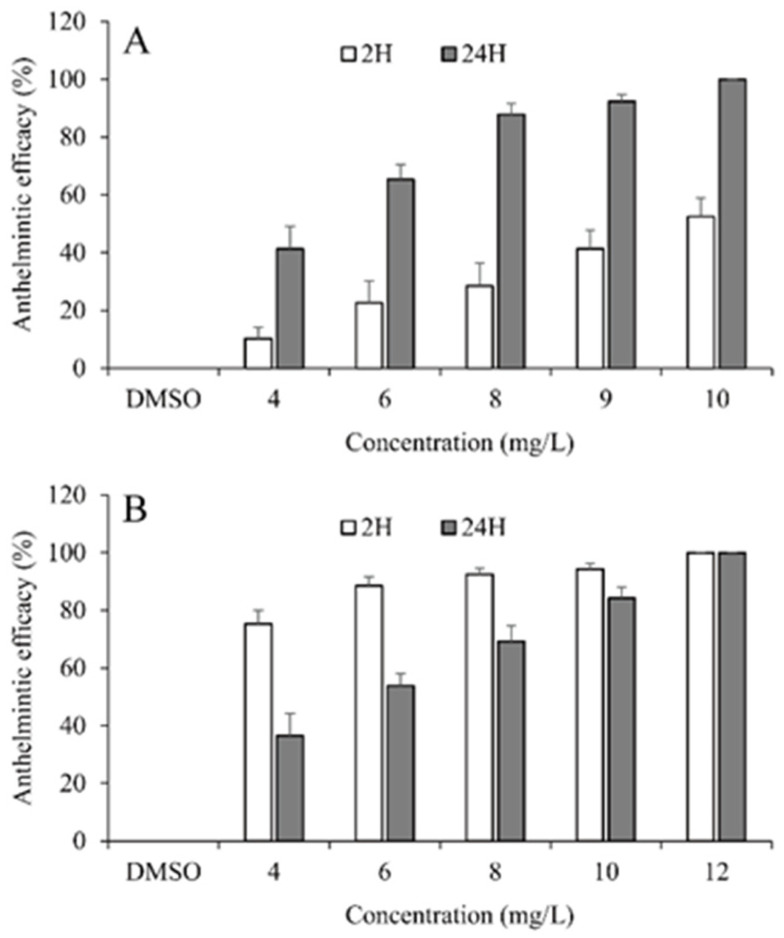
Anthelmintic efficacy of palmarosa oil (**A**) and curcuma oil (**B**) against *Gyrodactylus kobayashii* in vivo after 2- and 24-h exposure.

The results of in vivo anthelmintic efficacy of palmarosa oil and curcuma oil against *G. kobayashii* are presented in Figure 2 and Table 2. For palmarosa oil, anthelmintic activity increased with the bath concentration and exposure time and reached 100% anthelmintic efficacy at 10 mg/L after 24-h exposure; its EC_50_ values were 9.87 and 4.98 mg/L after 2- and 24-h exposure. However, in the case of curcuma oil, anthelmintic efficacy was found to increase with the bath concentration, but not with exposure time. Anthelmintic efficacy after 2-h exposure was higher than that after 24-h exposure at the same concentrations, except for the concentration of 12 mg/L, at which 100% anthelmintic efficacy was achieved after 2 and 24-h exposure. The EC_50_ values of curcuma oil were 3.48 and 5.72 mg/L after 2 and 24-h exposure, respectively. In the control group (0.03% DMSO), increased parasite load was observed, indicating no anthelmintic activity of 0.03% DMSO.

### 3.3. Acute Toxicity Tests against Goldfish

The results of acute toxicity tests of palmarosa oil and curcuma oil against goldfish are presented in Table 3. For palmarosa oil, the occurrence of fish death was observed at the concentration of 30 mg/L, and the number of dead fish increased with the bath concentration and exposure time; the mortality of goldfish reached 100% within 24 h at the concentration of 50 mg/L. The 24-h and 48-h LC_50_ of palmarosa oil against goldfish were 40.8 and 39.15 mg/L, respectively. As a measure of the relative safety or risk of the drug, the therapeutic index (TI, LC_50_/EC_50_) of these two EOs was also calculated and the TI value of palmarosa oil after 24-h exposure was 8.19. Similar results were observed for curcuma oil; no obvious toxicity to goldfish was observed at the concentration below 20 mg/L after a 48-h exposure, and the mortality of goldfish reached 100% within 24 h at 40 mg/L. The 24-h and 48-h LC_50_ of curcuma oil were 31.73 and 28.85 mg/L, respectively, and the TI value of curcuma oil after 24-h exposure was 5.54.

### 3.4. The Physiological and Histological Effects of EOs on Goldfish

**Figure 3:**
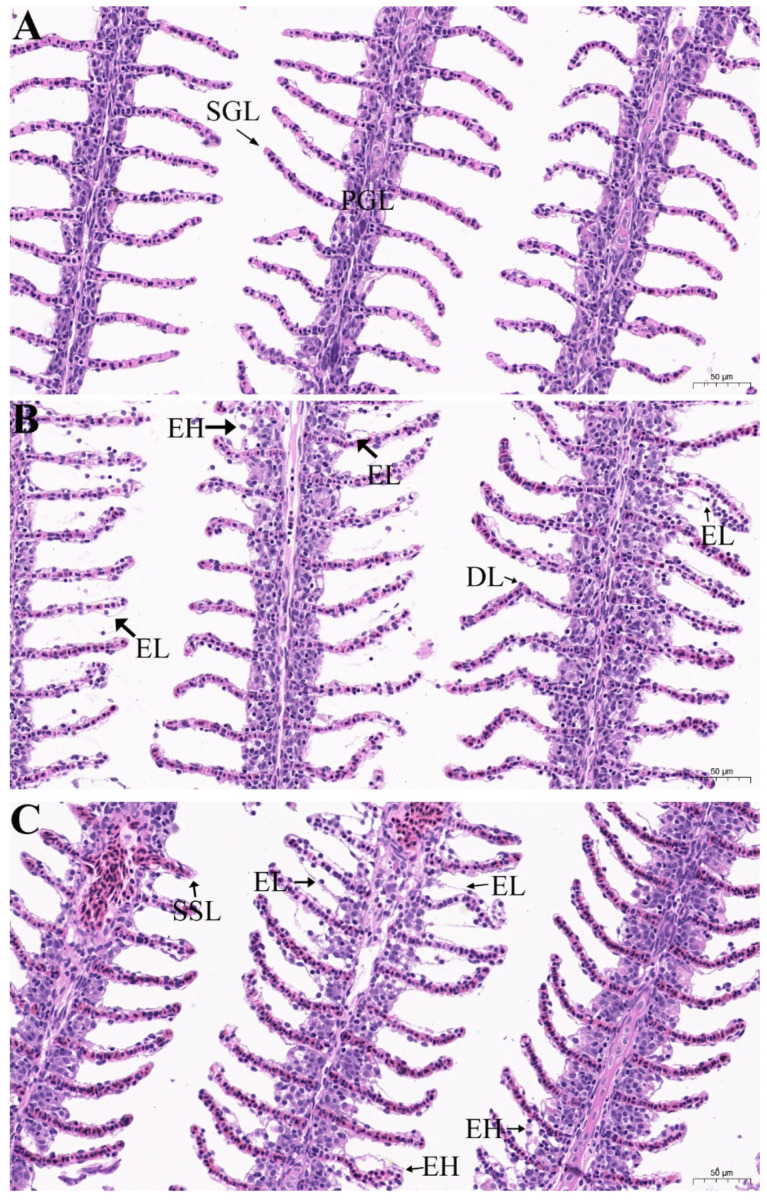
Histopathological changes in the gills of goldfish (*Carassius auratus*) after exposure to EOs. (**A**) Goldfish gills exposed to 0.2% DMSO for 96 h (control group), primary gill lamellae (PGL) and secondary gill lamellae (SGL); (**B**) goldfish gills exposed to 10.0 mg/L palmarosa oil for 96 h, epithelial hyperplasia (EH), epithelial lifting (EL), and deformed lamellae (DL); (**C**) goldfish gills exposed to 12.0 mg/L curcuma oil for 96 h, epithelial hyperplasia (EH), epithelial lifting (EL), and shortening of secondary lamellae (SSL).

As shown in Table 4, there was no significant difference in all hematological indexes measured in this study between different groups. Histological analysis after exposure to EOs is shown in Figure 3. The gills of the control group showed normal tissue structures with primary gill lamellae and secondary gill lamellae (Figure 3A). The gills in the exposed groups also displayed complete structures but with low to moderate histopathological alterations, including epithelial hyperplasia and lifting, deformed lamellae, and shortening of secondary lamellae (Figure 3B,C).

## 4. Discussion

Aquaculture is an important source of animal protein; however, the diseases caused by monogenean parasites may result in the retarded growth or high mortality of aquatic animals and represent a major threat to the development of aquaculture [24]. The environmental and health concerns caused by the use of chemical anthelmintics for disease control and the emergence of drug resistance have prompted researchers to search for effective alternatives [8,25]. Plant-derived and environment-friendly essential oils may be an ideal substitute, and many essential oils have been reported to be effective against monogenean [16,20,21]. Therefore, to search for safe and efficient anthelmintic agents for the control of monogenean infection, in this study, anthelmintic efficacy of 16 kinds of EOs was investigated, and the effects of the two EOs with superior anthelmintic activity, namely palmarosa oil and curcuma oil, on the survival of *G. kobayashii* and goldfish were also researched. The results from this study indicated that anthelmintic efficacy of palmarosa oil against *G. kobayashii* was in a time and dose-dependent manner. Curcuma oil showed an anesthetic effect on *G. kobayashii*, and its anthelmintic activity was dose-dependent rather than time-dependent in the concentration range tested in this study. Additionally, the two EOs showed weak toxicity to goldfish and had no serious physiological and histopathological influence on fish at the concentration of completely removing *G. kobayashii*.

Palmarosa oil, an essential oil extracted from the leaves and flowers of palmarosa grass, exhibited antifungal, nematocidal, and antioxidant properties [26,27,28]. In the present study, palmarosa oil displayed potent anthelmintic activity against *G. kobayashii* with a 24 h-EC_50_ value of 4.98 mg/L, and the in vitro assays indicated that therapeutic baths with a high concentration of this EO could cause more than 90% mortality of these worms within 8 hours. Similar results were reported by Nirmal et al. [29], who have shown that exposure to palmarosa oil causes paralysis and death of earthworms (*Pheretima posthuman*) and the high content of geraniol in the EO might be responsible for its anthelmintic activity. The high mortality of the soil worm (*Meloidogyne graminicola*) was also observed after exposure to palmarosa oil, which might be due to the presence of geraniol [27]. In addition, geraniol is the main bioactive compound of palmarosa oil, occupying 67.6–83.6% of the oil composition [30]. Therefore, it is speculated that geraniol might be the main active component against gyrodactylid monogenean. Moreover, palmarosa oil has been reported to be effective against *Aeromonas veronii* and *A. caviae*, potential pathogens of fish [31]. Obviously, due to its versatile properties, palmarosa oil not only repelled worms on fish but also effectively inhibited secondary infections with pathogenic bacteria caused by parasite infection. Thus, palmarosa oil might be a potential candidate agent to control monogenean infections in aquaculture.

In the current study, curcuma oil exhibited distinct anthelmintic properties against *G. kobayashii* in comparison with palmarosa oil. Unexpectedly, the in vivo assays indicated that anthelmintic efficacy against gyrodactylids after exposure for 24 h was not higher than that after exposure for 2 h. Therapeutic baths with curcuma oil for a longer time resulted in a decrease in anthelmintic efficacy. The reason for this unexpected result may be related to the life history of gyrodactylids. Unlike other monogeneans, gyrodactylids can continuously transmit between hosts during the whole life stages [4]. Gyrodactylids can survive off the host for several hours and have the ability to reinfect when they come into contact with a suitable host [32]. Therefore, it could be speculated that curcuma oil showed an anesthetic effect on gyrodactylids. Upon the initial exposure to curcuma oil, the anesthetized gyrodactylids detached from the host, resulting in an increase in anthelmintic efficacy. With the prolongation of exposure time, gyrodactylids gradually recovered from anesthesia and reinfected the host, thus leading to a decrease in anthelmintic efficacy. To our knowledge, it is first time reported here that curcuma oil had an anesthetic effect on monogenean, although previous studies have revealed that it also had an anesthetic effect on fish [33]. The main ingredients of this EO have been clarified [34], but the specific compounds responsible for the anesthetic effect are still unclear and need to be further studied.

Although the application of the anesthetic effect of curcuma oil to control monogenean infections faced various limitations in large-scale ponds, it could be used in some small-size or domestic farming systems that are convenient for water renewal. For example, some ornamental fish could be exposed to this EO for a short time to make the worms fall off from the host, and then the cultured water can be replaced to avoid reinfection. In addition, this EO could also be used to treat other monogenean infections, except for gyrodactylids. For these oviparous monogeneans, oncomiracidia hatched from eggs are the only infectious phase in the life cycle, and these larvae seek potential hosts and develop into adult worms on the host [35]. These adult worms shed from the host cannot swim freely and have almost no opportunity to reinfect the host [36]. Thus, baths with curcuma oil might be an effective strategy to control these oviparous monogeneans. Moreover, curcuma oil is also capable of killing gyrodactylids completely, which was supported by the results that all worms died within 4 h after exposure to 24 mg/L of this EO. This might be associated with the presence of curlone in this EO, which showed potent anti-trypanosomal activity [37]. Nevertheless, the content of this active ingredient in the EO is very low, accounting for only 5.15% [37]. Moreover, the major components in the essential oils were α-phellandrene, 1,8-cineole, α-zingiberene, *ar*-turmerone, α-turmerone, and β-turmerone, and the latter two active components have been reported to be responsible for the larvicidal activity of this EO on *Anopheles gambiae* [38,39]. Therefore, the observed anthelmintic activity against *G. kobayashii* in this study might partially be due to the aforementioned active ingredients in this EO, or a synergistic effect between these active ingredients. Collectively, curcuma oil showed anesthetic and anthelmintic effects on gyrodactylids and has the potential as an alternative agent for the treatment of *G. kobayashii* infections.

Therapeutic bath has been the conventional method to control fish ectoparasites, which exerted unexpected effects on the host and non-target organisms [40]. Although extensive studies have indicated that many EOs are beneficial to the health of fish [41], high concentration or long-term exposure to EOs still has adverse effects on fish (reviewed in [16]). Therefore, to evaluate the safety of the two EOs mentioned above, acute toxicity tests against goldfish and the effects on the hematology and histopathology of goldfish after exposure to EOs were investigated. Toxicological evaluation results revealed that palmarosa oil and curcuma oil had 48-h LC_50_ values of 39.15 and 28.85 mg/L, respectively. Moreover, the 24-h LC_50_ against goldfish of these two EOs was 8.19-fold and 5.54-fold higher than their corresponding EC_50_ against *G. kobayashii*, respectively. These results indicated a high safety of these two EOs. Hematological parameters are useful criteria for appraising the physiological alterations of fish [42]. In this study, no significant changes in the hematological parameters between the EOs-exposed groups and the control group were observed despite some fluctuations in some indicators between different groups. Additionally, no serious histopathological alterations were observed in the gills of goldfish after exposure to EOs at the concentrations of 100% effective against *G. kobayashii*. Moreover, these two EOs have been approved as food coloring or flavoring agents and recognized as “Generally Regarded as Safe” by the Food and Drug Administration (FDA) [43,44]. Overall, these two EOs are safe and had weak physiological and histopathological effects on goldfish at the concentrations tested in this study.

## 5. Conclusions

The development of an environment-friendly alternative for the control of monogenean infections can not only reduce the economic losses caused by these parasites but also ease public concerns about food safety and environmental pollution, which will contribute to the sustainable development of aquaculture. This study demonstrated that palmarosa oil exhibited potent anthelmintic activity against *G. kobayashii* in a time and dose-dependent manner. Meanwhile, curcuma oil showed anesthetic and anthelmintic effects on gyrodactylids. Moreover, these two EOs displayed weak toxicity and had no serious physiological and histopathological influence on goldfish. Therefore, palmarosa oil and curcuma oil could be potential candidates for the treatment of *G. kobayashii* infections. Nonetheless, the active compounds responsible for the anthelmintic and anesthetic effects should be identified and further verified.

## Figures and Tables

**Table 1:** Summary of tested essential oils used in the present study. AE, anthelmintic efficacy in vivo against *Gyrodactylus kobayashii* in goldfish (*Carassius auratus*); CAS, Chemical Abstracts Service; CAE, the concentration with the best anthelmintic efficacy; CFD, the concentration causing fish mortality.

Essential Oil	CAS	Source	Maximum Anthelmintic Efficacy (%)	CAE (mg/L)	CFD (mg/L)
Palmarosa oil	8014-19-5	*Cymbopogon martinii* (Roxb.) Wats.	100	10	30
Curcuma oil	8024-37-1	*Curcuma longa* Linn.	100	12	25
Cablin patchouli oil	8014-09-3	*Pogostemon cablin* (Blanco) Benth. *	100	10	10
Zedoary oil	/	*Curcuma zedoaria* (Christm.) Rosc.	100	15	20
Rue oil	8014-29-7	*Ruta graveolens* Linn. *	100	25	40
Tea tree oil	68647-73-4	*Melaleuca alternifolia* Cheel	100	40	50
Neem oil	/	*Melia azedarace* Linn.	100	40	45
Cassia oil	8015-96-1	*Cinnamomum cassia* Presl	94.57	14	14
Eucalyptus oil	8000-48-4	*Eucalyptus globulus* Labill. *	90.84	100	100
Clove oil	8000-34-8	*Syzygium aromaticum* (L.) Merr. Et Perry	45.06	16	16
Clove leaf oil	8015-97-2	*Syzygium aromaticum* (L.) Merr. Et Perry	36.02	18	18
Origanum oil	8007-11-2	*Origanum vulgare* Linn.	23.36	10	10
Anise oil	8007-70-3	*Pimpinella anisum* Linn.	21.92	10	10
Peppermint Oil	68917-18-0	*Mentha sachalinensis* (Briq. ex Miyaabe et Miyake) Kudo *	10.45	40	40
Fennel Oil	8006-84-6	*Foeniculum vulgare* Mill.	10.1	40	40
Lemon oil	84929-31-7	*Citrus limon* (L.) Burm. F.	5.62	10	10

Note: “/” indicated CAS number of the essential oil was not found; “*” indicated the essential oil might be extracted from other plants of the same genus.

**Table 2:** Anthelmintic efficacy (EC_50_ and EC_90_) of palmarosa oil and curcuma oil against *Gyrodactylus kobayashii* after 2- and 24-h exposure.

Essential Oil	Exposure Time (h)	EC_50_ (95%CI, mg/L)	EC_90_ (95%CI, mg/L)
Palmarosa oil	2	9.87 (9.24–10.72)	15.4 (13.92–17.7)
	24	4.98 (4.11–5.66)	8.07 (7.3–9.22)
Curcuma oil	2	3.48 (0–5.21)	6.7 (4.97–10.84)
	24	5.72 (4.14–6.86)	9.34 (8.02–12.17)

Note: EC_50_ and EC_90_, 50% effective concentration; 95% CI, 95% confidence interval.

**Table 3:** Acute toxicity of palmarosa oil and curcuma oil against goldfish after 24 and 48 h of exposure. SD, standard deviation; LC_50_, 50% lethal concentration; 95% CI, 95% confidence interval.

Essential Oil	Concentration (mg/L)	No. of Fish/Tank	No. Dead Fish (mean ± SD)		LC_50_ (95%CI, mg/L)	
			24 h	48 h	24 h	48 h
Palmarosa oil	0	10	0	0	40.8 (38.26–43.43)	39.15 (36.36–41.86)
	30	10	0	0.33 ± 0.58		
	35	10	1.67 ± 1.58	2.67 ± 0.58		
	40	10	4.33 ± 0.58	5.67 ± 1.15		
	45	10	7.33 ± 0.58	8.0 ± 1.0		
	50	10	10	10		
Curcuma oil	0	10	0	0	31.73 (29.2–34.51)	28.85 (26.06–31.5)
	20	10	0	0		
	25	10	1.67 ± 0.58	3.57 ± 0.58		
	30	10	3.0 ± 1.0	5.33 ± 0.58		
	35	10	6.67 ± 0.58	8.33 ± 0.58		
	40	10	10	10		

**Table 4:** The hematological indicators of goldfish (*Carassius auratus*) after exposure to palmarosa oil, curcuma oil, and 0.2% DMSO for 96 h (*n* = 5; mean ± SD). Different letter in the same rows indicates differences (*p* < 0.05) (one-way ANOVA followed by Duncan post-test). RBC, red blood cell; Hb, hemoglobin concentration; HCT, hematocrit; MCV, mean corpuscular volume; MCH, mean corpuscular hemoglobin; MCHC, mean corpuscular hemoglobin concentration; PLT, platelet.

Treatment	0.2% DMSO	Palmarosa Oil	Curcuma Oil
RBC (10^12^/L)	0.98 ± 0.12 ^a^	0.94 ± 0.15 ^a^	1.16 ± 0.28 ^a^
Hb (g/L)	105 ± 18.01 ^a^	103.6 ± 10.24 ^a^	109.2 ± 13.08 ^a^
HCT (%)	16.18 ± 2.32 ^a^	15.12 ± 2.24 ^a^	15.52 ± 1.58 ^a^
MCV (fL)	179.2 ± 8.09 ^a^	177.96 ± 8.9 ^a^	176.16 ± 12.25 ^a^
MCH (pg)	127.94 ± 4.9 ^a^	128.06 ± 12.64 ^a^	133.5 ± 11.9 ^a^
MCHC (g/L)	667.8 ± 62.78 ^a^	747.4 ± 87.99 ^a^	731 ± 50.4 ^a^
PLT (10^9^/L)	47.8 ± 5.89 ^a^	58 ± 17.25 ^a^	43.8 ± 18.75 ^a^

## Data Availability

The datasets used and analyzed during the current study are available from the corresponding author on reasonable request.
